# Albany Medical College

**Published:** 1859-05

**Authors:** 


ALBANY MEDICAL COLLEGE.—As will be seen in our advertising sheet, the annual course of lectures in this institution will commence, as formerly, on the first Tuesday of September. But one term will be held yearly.

